# Supplementary material for: A qualitative study on the perspectives of prenatal breastfeeding educational classes in Ireland: Implications for maternal breastfeeding decisions
Source: PLoS One. 2024 Dec 18;19(12):e0315269. doi: 10.1371/journal.pone.0315269 (PMC11654992; doi:10.1371/journal.pone.0315269)
Supplement: S1 Table — (DOCX) [file pone.0315269.s001.docx]

**S1 Table: COREQ checklist.**

| **COREQ CHECKLIST** | **Reported in Manuscript** |
| --- | --- |
| **Domain 1: Research team and reflexivity** |  |
| *Personal Characteristics* |  |
| 1. Interviewer/facilitator: Which author/s conducted the interview or focus group? | Yes. P9-10 |
| 2. Credentials: What were the researcher’s credentials? | Yes. This is in the Title Page |
| 3. Occupation: What was their occupation at the time of the study? Registered Nurse/Midwife | Yes. This is in the Title Page |
| 4. Gender: Was the researcher male or female? | Yes. This is in the Title Page |
| 5. Experience and training: What experience or training did the researcher have? | Yes. P12 |
| *Relationship with participants* |  |
| 6. Relationship established: Was a relationship established prior to study commencement?  Yes, not a formal relationship, some participants may have known the names of, and met some of the research team over the course of their careers. | Yes, a formal relationship was established with participants before the study began. This involved official communication and consent processes to ensure clarity and trust. |
| 7. Participant knowledge of the interviewer: What did the participants know about the researcher? | No |
| 8. Interviewer characteristics: What characteristics were reported about the interviewer/facilitator? | Yes. This is in the Title Page |
| **Domain 2: Study design** |  |
| *Theoretical framework* |  |
| 9. Methodological orientation and Theory: What methodological orientation was stated to underpin the study? | Yes. P8 |
| *Participant selection* |  |
| 10. Sampling: How were participants selected? | Yes. P8-10 |
| 11. Method of approach: How were participants approached? | Yes. P9-10 |
| 12. Sample size: How many participants were in the study? | Yes. P10 |
| 13. Non-participation: How many people refused to participate or dropped out? Reasons? | No. This information was not collected. |
| *Setting* |  |
| 14. Setting of data collection: Where was the data collected? | Yes. P8-9 |
| 15. Presence of non-participants: Was anyone else present besides the participants and researchers? | No, no one else was present. |
| 16. Description of sample: What are the important characteristics of the sample? | Yes. P9, P15-18. |
| *Data collection* |  |
| 17. Interview guide: Were questions, prompts, guides provided by the authors? Was it pilot tested? | Yes. P10 |
| 18. Repeat interviews: Were repeat interviews carried out? If yes, how many? | No |
| 19. Audio/visual recording: Did the research use audio or visual recording to collect the data? | Yes, P8 and 10 |
| 20. Field notes: Were field notes made during and/or after the interview or focus group? | Yes. P11 and 14. |
| 21. Duration: What was the duration of the interviews or focus group? | Yes. P10 |
| 22. Data saturation: Was data saturation discussed? | Yes. P12 |
| 23. Transcripts returned: Were transcripts returned to participants for comment and/or correction? | Yes, participants were allowed to review their transcripts; only two accepted them and were satisfied with the content and accuracy. |
| **Domain 3: Analysis and findings** |  |
| *Data analysis* |  |
| 24. Number of data coders: How many data coders coded the data? | Yes. P12 |
| 25. Description of the coding tree: Did authors provide a description of the coding tree? | Yes. P20-21 |
| 26. Derivation of themes: Were themes identified in advance or derived from the data? | Derived from the data. P20-21 |
| 27. Software: What software, if applicable, was used to manage the data? | Yes. Version 14; QSR, 2024. P 12. |
| 28. Participant checking: Did participants provide feedback on the findings? | Yes. Feedback was provided in the unpublished final phase of this study. |
| *Reporting* |  |
| 29. Quotations presented: Were participant quotations presented to illustrate the themes/findings? Was each quotation identified? e.g. participant number | Yes. P22-50, and in Supplementary File 6 |
| 30. Data and findings consistent: Was there consistency between the data presented and the findings? | Yes. P22-50 |
| 31. Clarity of major themes: Were major themes clearly presented in the findings? | Yes. P22-50 |
| 32. Clarity of minor themes: Is there a description of diverse cases or discussion of minor themes? | Yes, within the significant themes P21-50 |
