# Supplementary material for: A qualitative study on the perspectives of prenatal breastfeeding educational classes in Ireland: Implications for maternal breastfeeding decisions
Source: PLoS One. 2024 Dec 18;19(12):e0315269. doi: 10.1371/journal.pone.0315269 (PMC11654992; doi:10.1371/journal.pone.0315269)
Supplement: S2 Table — (DOCX) [file pone.0315269.s002.docx]

**S2 Table: GRIPP2 short form *(PPI=patient and public involvement).***

| **GRIPP2 CHECKLIST** | **Item** | **Reported on page No** |
| --- | --- | --- |
| **Section and Topic** |  |  |
| 1. **Aim**   Report the aim of the study. | To ensure the collaborative involvement of patients as research partners at all stages of the GRIPP2 development process. | Yes. Page 10 |
| 1. **Methods**   Provide a clear description of the methods used for PPI in the study. | The PPI group, comprising two postnatal mothers, a lactation consultant, and a midwife experienced in prenatal breastfeeding education, provided essential input during the study design phase. Their first-hand experience and insights ensured the research questions and objectives were relevant and meaningful to the target population. They were solicited for their expert counsel, tasked with providing critical feedback on the interview topic guide and engaged in collaborative efforts regarding the recruitment process. | Yes. Page 10 |
| 1. **Study results**   Outcomes—Report the results of PPI in the study, including both positive and negative outcomes | Their input was invaluable in refining the research questions to make them more relevant and specific to the experiences and needs of the target population. This helped obtain more focused and meaningful data. The PPI contributors provided feedback on the interview guides, ensuring the questions were straightforward, culturally sensitive, and appropriate for the participants. This led to more effective and comfortable data collection sessions. | Yes. Page 10 |
| 1. **Discussion and conclusions**   Outcomes—Comment on the extent to which PPI influenced the study overall. Describe the positive and negative effects. | Integrating patient and public involvement (PPI) in this study proved exceptionally effective, significantly shaping several pivotal aspects. This efficacy can be ascribed to multiple factors, including the researchers' adeptness in engaging patient partners in their research endeavours. Robust procedural frameworks were established, ensuring the engagement of patient partners from the study's inception, thereby enabling them to shape and contribute to the research process meaningfully. This collaborative dynamic was instrumental in enhancing the positive impact of PPI. The pre-existing relationships with patient partners and participants of the collaborative consensus event furnished essential contextual grounding for the embedded PPI. Nevertheless, certain limitations were observed: the feedback window between researchers and PPI was constrained, and coordinating mutually convenient meeting times presented challenges. Future studies of a similar nature might mitigate these limitations by more effectively scheduling such meetings. | Yes. Page 10 |
| 1. **Reflections/critical perspective**   Critical perspective—Comment critically on the study, reflecting on the things that went well and those that did not, so others can learn from this experience | This study offers a robust framework for integrating Patient and Public Involvement (PPI), and a critical reflection of this process elucidates a nuanced understanding of its successes and challenges, providing valuable insights for future research endeavours. A noteworthy achievement of PPI in this context was the enhanced relevance and credibility of the findings. Including perspectives from midwives, lactation consultants, and mothers furnished essential contextual grounding, anchoring the study with authentic, real-world experiences. However, the study encountered several challenges that underscore areas necessitating improvement. The primary challenge was the constrained feedback period between researchers and patient partners. From these reflections, several recommendations for future studies become apparent. Extending the feedback period would facilitate more thoughtful and comprehensive contributions from patient partners. Additionally, enhancing scheduling flexibility through digital tools could better accommodate diverse availabilities, ensuring consistent engagement. | Yes. Page 10 |
