# Supplementary material for: A qualitative study on the perspectives of prenatal breastfeeding educational classes in Ireland: Implications for maternal breastfeeding decisions
Source: PLoS One. 2024 Dec 18;19(12):e0315269. doi: 10.1371/journal.pone.0315269 (PMC11654992; doi:10.1371/journal.pone.0315269)
Supplement: S3 Table — (DOCX) [file pone.0315269.s003.docx]

**S3 Table: Interview topic guides.**

| **Experiences of providing breastfeeding education** |
| --- |
| 1. How would you describe the mother’s engagement in your breastfeeding education classes? |
| 1. How are the breastfeeding education classes structured to cater to the individual needs of each mother? |
| 1. How do you evaluate the mother’s understanding of breastfeeding education provided? |
| 1. How do you identify or recognize mothers who are having difficulties engaging in your class, and what do you do to support them? |
| 1. Do you think these classes influence mothers to breastfeed? Please explain. |
| 1. What types of practical advice do you provide during prenatal breastfeeding educational classes? |
| 1. Do you provide additional support to the mothers during prenatal breastfeeding education classes? |
| **Breastfeeding knowledge, skills, and competence in providing breastfeeding education.** |
| 1. What essential skills do you use to help mothers achieve their breastfeeding goal? |
| 1. What resources do you use to assist mothers with breastfeeding challenges? |
| 1. What do you think is the most critical skill when providing/supporting mothers with breastfeeding education? |
| 1. What misconceptions are expressed by the women about breastfeeding, and how do you address these misconceptions? |
| **Improvement of Breastfeeding educational classes for optimum breastfeeding uptake** (*What can be done better, and what are the areas for improvement)* |
| 1. If you were to design, update/change the current breastfeeding educational classes what would you recommend? |
| 1. What changes or suggestions do you think these classes need to improve engagement and accessibility for mothers? |
| 1. What educational support would you need to further enhance these breastfeeding classes? |
| **Consistency of Ongoing Support/Follow-Up.** |
| 1. What information is available for mothers on how to access breastfeeding support services in their local community? |
| **Socio-Cultural/Culturally Competent Support for Breastfeeding** |
| 1. Describe your experience of meeting mother’s cultural needs and beliefs while educating them on best breastfeeding practices. |
| 1. What are your views on women breastfeeding in public? |
| 1. Do the breastfeeding classes prepare mothers for breastfeeding in public? |
| 1. Do you think the topic of breastfeeding in public should be explored further in the classes? |
| 1. What are your thoughts on breastfeeding as the best method for infant feeding? |
| Is there anything else you would like to add and say etc…?  ***Thank you for participating*** |

1. **Interview topic guide for lactation consultants/midwives:**
2. **Interview topic guide for post-natal mothers:**

| **Perception/Experiences of Prenatal Breastfeeding Classes** *(Mother’s perspectives of prenatal breastfeeding educational classes)* |
| --- |
| 1. How did you find the prenatal breastfeeding educational classes? please describe your overall experience. |
| 1. What were the main skills/knowledge/techniques you learned from the breastfeeding classes? What was beneficial, and what did not go so well? |
| 1. Were you able to understand all the information in the breastfeeding classes? |
| 1. How did the information you received in the breastfeeding education classes improve your breastfeeding knowledge? |
| 1. Describe how the information received in the breastfeeding education classes prepared or supported you to breastfeed and to continue breastfeeding. |
| **Support, competency skills and training requirements in the designing and delivering of prenatal breastfeeding education. (What is required in the prenatal breastfeeding education classes)** |
| 1. What other topics would you have benefitted from that were not included in the breastfeeding education classes? (i.e., skills, content, support, info, material, resources, links with others, etc.) |
| 1. How did you find the breastfeeding educators? (i.e., knowledge, support, teaching skills, approaches, engagement). |
| 1. Can you describe/remember anything significant they made that impacted your decision to breastfeed? |
| 1. Were you provided opportunities to evaluate or provide feedback after the breastfeeding education sessions? |
| **Improvement of Breastfeeding educational classes for optimum breastfeeding uptake** (*What can be done better, and what are the areas for improvement)* |
| 1. If you were to design, update/change the current breastfeeding educational classes what would you recommend? |
| 1. What changes or suggestions do you think these classes need to improve engagement and accessibility for mothers? |
| 1. What educational support would you need to further enhance these breastfeeding classes? |
| **Culture and Attitudes towards Breastfeeding.** |
| 1. How would you describe the attitudes towards breastfeeding in your area/social group? |
| 1. Do you think the women you know/socialise with will naturally choose to breastfeed? |
| 1. What are your views on women breastfeeding in public? |
| 1. Did the breastfeeding classes prepare you for breastfeeding in public? |
| 1. Do you think this area should be explored in the breastfeeding classes? |
| 1. Describe the attitudes and encouragement you received from your partner, other family members and friends with breastfeeding |
| 1. Was Partner involvement discussed in the breastfeeding class? |
| **Intention to continue with Breastfeeding.** |
| 1. Did the breastfeeding classes equip you to deal with breastfeeding challenges? |
| 1. Did you feel confident about raising any issues in the breastfeeding classes? |
| 1. Did you feel supported/encouraged to raise these issues? |
| 1. If you encounter any breastfeeding problems, would you seek support or discontinue breastfeeding? |
| **Women’s Preferred method of breastfeeding education class.** |
| 1. Given the option of online, face-to-face, webinar, or group/individualized breastfeeding educational class, what would be your preferred method and why? |
| 1. What do you think of the duration of the breastfeeding classes? |
| Is there anything else you would like to add and say etc…?  ***Thank you for participating*** |
