# Supplementary material for: A qualitative study on the perspectives of prenatal breastfeeding educational classes in Ireland: Implications for maternal breastfeeding decisions
Source: PLoS One. 2024 Dec 18;19(12):e0315269. doi: 10.1371/journal.pone.0315269 (PMC11654992; doi:10.1371/journal.pone.0315269)
Supplement: S4 Table — (DOCX) [file pone.0315269.s004.docx]

| **Items** | ***Agree, (%)*** | ***Neutral, (%)*** | ***Disagree (%)*** |
| --- | --- | --- | --- |
|  |  |  |  |
| 1. The benefit of breastfeeding lasts only as long as the baby is breastfed. | 11 (55%) | 3 (15%) | 6 (30%) |
| 1. Formula feeding is more convenient than breastfeeding. | 9 (45 %) | 5 (25%) | 6 (30%) |
| 1. Breastfeeding increases mother-infant bonding. | 17 (85%) | 3 (15%) | 0 (0%) |
| 1. Breastmilk is lacking in iron. | 4 (20%) | 9 (45%) | 7 (35%) |
| 1. Formula-fed babies are more likely to be overfed than breastfed babies. | 10 (50%) | 7 (35%) | 3 (15%) |
| 1. Formula feeding is the better choice if the mother plans to go back to work. | 16 (80%) | 3 (15%) | 1 (5%) |
| 1. Mothers who formula feed miss one of the great joys of motherhood. | 2 (10%) | 2 (10%) | 16 (80%) |
| 1. Women should not breastfeed in public places such as markets, worship centres, offices or restaurants. | 12 (60%) | 5 (25%) | 3 (15%) |
| 1. Breastfed babies are healthier than formula-fed babies. | 8 (40%) | 5 (25%) | 7 (35%) |
| 1. Breastfed babies are more likely to be overfed than formula-fed babies. | 3 (15%) | 6 (30%) | 11 (55%) |
| 1. The father feels left out if the mother breastfeeds. | 4 (20%) | 3 (15%) | 13 (65%) |
| 1. Breastmilk is the ideal food for infants. | 17 (85%) | 3 (15%) | 0 (0%) |
| 1. Breastmilk is more easily digested than formula. | 11 (55%) | 6 (30%) | 3 (15%) |
| 1. Formula is as healthy for an infant as breastmilk. | 13 (65%) | 4 (20%) | 3 (15%) |
| 1. Breastfeeding is more convenient than formula feeding. | 3 (15%) | 5 (25%) | 12 (60%) |
| 1. Breastfeeding is cheaper than formula feeding. | 18 (90%) | 2 (10%) | 0 (0 %) |
| 1. A Mother who occasionally drinks alcohol should not breastfed. | 15 (75%) | 3 (15%) | 2 (10%) |

**S4 Table: Iowa Infant Feeding Attitude Scale (IIFAS).**
