# Supplementary material for: A qualitative study on the perspectives of prenatal breastfeeding educational classes in Ireland: Implications for maternal breastfeeding decisions
Source: PLoS One. 2024 Dec 18;19(12):e0315269. doi: 10.1371/journal.pone.0315269 (PMC11654992; doi:10.1371/journal.pone.0315269)

**S5 Table: HSE Self-Assessment Competency Framework for Breastfeeding Support.**

| HSE Self-Assessment Competency Framework for Breastfeeding Support | LC  01 | LC 02 | LC  03 | GCO3 | GC  01 | GC  02 | RC  01 | RC  02 | CC  01 | CC  02 |
| --- | --- | --- | --- | --- | --- | --- | --- | --- | --- | --- |
| 1. Explaining The Importance of Breastfeeding. | ✓ | ✓ | ✓ | ✓ | ✓ | ✓ | ✓ | ✓ | ✓ | ✓ |
| 1. Identifying Practices that Support or Could Harm Breastfeeding. | ✓ | ✓ | ✓ | ✓ | ✓ | ✓ | ✓ | ✓ | ✓ | ✓ |
| 1. Assisting With Breastfeeding Techniques. | ✓ | ✓ | ✓ | ✓ | ✓ | ✓ | ✓ | ✓ | ✓ | ✓ |
| 1. Recognizing Feeding Cues and Challenges. | ✓ | ✓ | ✓ | ✓ | ✓ | ✓ | ✓ | ✓ | ✓ | ✓ |
| 1. Guiding on Manual Expression and Use of Breast Pumps. | ✓ | ✓ | ✓ | ✓ | ✓ | ✓ | ✓ | ✓ | ✓ | ✓ |
| 1. Identifying and Taking Important Actions in Potential Breastfeeding Problems (e.g., Mastitis, Breast Trauma). | ✓ | ✓ | ✓ | ✓ | ✓ | ✓ | ✓ | ✓ | ✓ | ✓ |
| 1. Providing Information on Feeding Expressed Breast Milk | ✓ | ✓ | ✓ | ✓ | ✓ | ✓ | ✓ | ✓ | ✓ | ✓ |
| 1. Identifying Practices that Support Breastfeeding (e.g., skin-to-skin, early initiation, rooming-in, feeding in response to infant’s needs). | ✓ | ✓ | ✓ | ✓ | ✓ | ✓ | ✓ | ✓ | ✓ | ✓ |
| 1. Assisting The Mother to Acquire Skills to Position and Latch to The Breast for Adequate feeding. | ✓ | ✓ | ✓ | ✓ | ✓ | ✓ | ✓ | ✓ | ✓ | ✓ |
| 1. Knowing When to Access Further Help. | ✓ | ✓ | ✓ | ✓ | ✓ | ✓ | ✓ | ✓ | ✓ | ✓ |


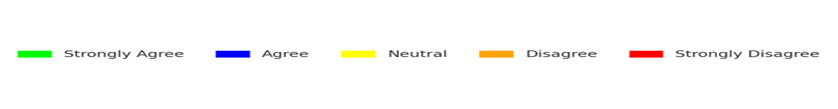

Supplement: S5 Table — (DOCX) [file pone.0315269.s005.docx]
