# Supplementary material for: A qualitative study on the perspectives of prenatal breastfeeding educational classes in Ireland: Implications for maternal breastfeeding decisions
Source: PLoS One. 2024 Dec 18;19(12):e0315269. doi: 10.1371/journal.pone.0315269 (PMC11654992; doi:10.1371/journal.pone.0315269)
Supplement: S6 Table — (DOCX) [file pone.0315269.s006.docx]

| **S6 Table: Illustrative quotations presented by theme and sub-themes.** | |
| --- | --- |
| **Theme Title** | **Illustrative Quotes** |
| **Theme One**  From Idealization to Informed Reality: Transforming Prenatal Breastfeeding Guidance. | ***POSTNATAL MOTHER’S QUOTES***  *In some ways, yes. They did a good job of covering the basics and telling me what to expect. But I wish there had been more focus on the problems that can happen and how to deal with them" (MC01).*  *Yeah. That’s why it should be taken as an important topic. But I didn’t really see that. They were just glossed over. I mean who doesn’t know that breast is best, we all know that there’s no denying that. So, I mean, I didn’t really get that feeling that they took the challenges seriously” (MC03).*  *It's clear they were trying to promote breastfeeding, and they provided good resources and support for it. But I think they need to give equal attention to the challenges. Make sure mums-to-be are fully prepared for the journey ahead, you know?" (ML01).*  *“Ah, yes, there were. The advice on how to breastfeed was grand, but I felt they left us a bit at sea about the actual challenges of breastfeeding. Straight after I got home, I was struggling with not enough milk and painful nipples, and I felt completely unprepared for it. It's like those issues were barely touched on in the class, leaving me feeling quite let down, to be honest” (MC01).*  *“There were, indeed, a few items. For instance, like I just told now the classes emphasized the advantages of breastfeeding, while ignoring the difficulties. I believe it is essential to be aware of these as well. Apart from that as well, I needed a lot of emotional and mental support because I was constantly under pressure to do everything right. Sometimes I got so overwhelmed and all I did was just cry” (ML05).*  *“Well, breastfeeding can be very emotional, can't it? With me first, I felt a lot of pressure to get it right, and when it didn’t work out, I felt like I was failing' as a mother. I was hoping' the class would address those feelings, you know, reassure mothers that it’s okay to feel overwhelmed or upset, and give strategies for dealing' with those emotions. But the class was more focused on all the good stuff and all the easy stuff, which is grand, but I was looking for a bit more”* *(MC05).*  ***HEALTHCARE PROFESSIONAL’S QUOTES (LACTATION CONSULTANTS /MIDWIVES).***  *Oh, it's been a challenge, for sure. The pandemic has thrown us all a curveball. Moving online, while necessary, has definitely made it harder to gauge how the mums are feeling and to offer them immediate support. We've lost a bit of the communal feeling, the shared experiences. But on the flip side, it has made the classes more accessible to some. We're learning, adapting. We want to use technology to better support our mums emotionally, not just educationally” (RC02).*  *“Moving online was a bit of a hurdle, to be sure. It's been tricky to replicate the warmth and personal touch of our in-person sessions in a virtual format. We couldn't offer the same level of comfort or read the room as we used to, and mums missed out on forming those natural supports with other mums. It's made us realize more than ever how vital the emotional and psychological aspects of breastfeeding support are” (GC01).*  *Well, moving online was a necessary step, wasn't it? But, it's been a double-edged sword. On one hand, we've been able to reach mothers who might not have been able to attend in person. On the other, the personal touch, that face-to-face interaction, has suffered. It's harder to create a warm, supportive environment over a screen. The nuances of reassurance, understanding the emotional cues – it's challenging. This has made it difficult for some mothers to fully express their frustrations and doubts, and for us to address them as effectively as we'd like” (CC01).* |
| **Subtheme 1.1.**  Impact of Unmet Breastfeeding Educational Expectations on Breastfeeding Decision.  **Subtheme 1.2**  The Psychological Impact of Encountering Unexpected Challenges After an Idealized Portrayal of Breastfeeding | *“It was a tough one, really. I felt prepared after the class, and I did. But then, when the real challenges hit, I was like, "What now?" They didn’t cover half of it, like how painful it can be, which left me feeling a bit stranded. It was a harsh lesson that there’s more to it than just the good bits they tell ya. Those early days tested me faith in continuing with breastfeeding. The class set me up with expectations that just didn’t square with what I faced, making the whole thing more daunting than I ever imagined” (MG02).*  *"It left me feeling' quite anxious, you know? I was wondering' if I’d be able to handle it especially as a new mom or if I was the only one struggling'. It’s like I was set up to expect this beautiful, natural thing, but then hit with the reality that it’s not always a walk in the park. Made me question if I was ready or even capable, which was really frustrating" (MC05).*  *“Breastfeeding is not just a physical act but also an emotional journey. With my first child, I struggled with everything; it was a disaster. I struggled with inadequacy and worry when things weren’t going smoothly. There's a lot of pressure on mums to get it right, and it can be very isolating when it's not going well” (MR03).*  *“For example, I wish they had discussed the mental and emotional obstacles of breastfeeding, like postpartum depression and anxiety, in greater detail. I personally labored with these issues, so I think that additional guidance and assistance would have been beneficial" (MG01).*  *“To be honest, that's a fair critique. We've been so wrapped up in the practical side of things we've maybe lost sight of the emotional journey of breastfeeding. It’s clear we’ve got a bit to learn about balancing the physical and emotional sides of breastfeeding support” (LC03).*  *“The move to online classes was a challenge, for sure. It made it harder to create that warm, supportive environment we strive for. We couldn't read the room the same way or offer comfort in person and we acknowledge how isolating this might be for the mothers sitting behind the screens listening to us. Plus, mums didn’t have the chance to form those in-person connections with other mums, which can be a huge source of support. We’ve realized that emotional support and understanding are just as important as the physical aspects of breastfeeding" (CC02).*  *“Sure, we're quite pleased with our program, but we know there's always space to make it better. Taking on board what we are discussing now, we aim to take the emotional sides of breastfeeding seriously and beef up our online support for a bit more tailored guidance. We're also thinking hard on how to better ready the mums for those unexpected challenges, making sure they're feeling completely backed up on their breastfeeding decisions” (RC02).* |
| **Theme Two**  Integrating Interactivity and Personalization for Enhanced Maternal Engagement. | ***POSTNATAL MOTHER’S QUOTES***  *“Well, to be fair, it was good on the basics. But, ye know, every mam has her own journey. I was looking' for something a wee bit more tailored to me own situation. Something more engaging that would let us mams share our stories and learn from each other in a more personal way” (MR02).*  *“But there's room for improvement. The class was a bit one-size-fits-all. I was hoping' for more opportunities to ask questions and get answers relevant to me own concerns. More interactive activities would've been nice, too, to really feel engaged and part of it all” (ML05).*  *“The format was alright, but I was hoping for something a bit more engaging. The information was grand, but it was a lot of listening and not as much interacting as I'd have liked. I was expecting more of a two-way conversation, where I could ask questions and get immediate feedback tailored to my own situation” (MC01).*  *“As mothers, we've all got different experiences, different concerns, different situations. And while the information provided was top notch, there was very little room for us to express our individual perspectives. It's important to feel heard, especially as a mother” (MG05).*  *“The format was informative, but if I'm honest, I was hoping for a bit more engagement. The content was solid, covering all the basics of breastfeeding, which was great. However, I felt the delivery was a bit impersonal. It was mostly watching presentations and listening to lectures. I had hoped for more interactive elements, like live discussions or even practice sessions that could have provided a more hands-on learning experience” (Mother L01).*  *“That's one area they could certainly improve. It would have been nice to have an anonymous survey or something of the sort, so that we could have shared our thoughts on the class without feeling embarrassed or singled out” (MG02).*  *“You see, breastfeeding can be a very personal and sometimes sensitive topic. Some of us might have had issues or concerns we were a bit embarrassed to voice out loud, especially in a group setting. Having a way to share those thoughts anonymously could really help the educators understand where they might need to adjust the class to better meet our needs. It's about feeling safe to share your true thoughts, you know?” (M R03).*  *“Yeah, there is one more thing I'd like to mention. The online class was convenient for me, I can see how it might not be the best option for everyone. Some expectant mothers might prefer face-to-face classes to have more personalized interactions and immediate answers to their questions. So, having a variety of options available, like both virtual and in-person classes, would be beneficial to cater to different preferences and needs” (ML03).*  *“Yes, I do think the virtual setting had an impact. I mean the midwives were knowledgeable about breastfeeding, it was sometimes challenging to ask questions or seek clarification during online sessions. It's just not the same as being in a physical classroom where you can easily interact with the instructor” (ML01).*  *“Well, they tried with the polls, which was a bit of craic, but it still didn't feel like enough. I was hoping' for more interactive bits, maybe breakout rooms where we could chat in smaller groups, or even one-on-one time with the lactation experts. Something to make it feel more personal and less like just watching a video?” (MC04).*  *“The class had loads of useful info, sure, but wanted a bit of a natter, you know? The quizzes were a nice attempt at making it interactive, but it still felt a bit flat. I was looking for a platform where me and the other mums could share a bit more personally, engage with the content on a deeper level” (MR04).*  *“When you're in a room with others, you can kinda feel the energy, ask questions directly, and get immediate feedback. Online, even with the quizzes and polls, it didn't feel engaging. I was hoping for more of a back-and-forth conversation, you know that kind of way” (ML01).*  *“I’d say, try to make the online classes more interactive. Use technology to your advantage, like polls, like question and answer, smaller rooms, I believe you can do that now. I mean create smaller groups within the class you know, stuff like that. And maybe check in with the mums attending, see if they have questions or need to discuss anything. Just because it’s virtual doesn’t mean it can’t be personal and engaging” (MR01).*  *"I'm reckoning' they could do with more of allowing the mothers to ask questions, I don’t like typing in my questions in the chat box, you know? Maybe get ourselves into smaller group chats where we can have the craic more openly and nab some real-time feedback. And what about a few personalized advice sessions, eh?” (MC01).*  *“Well, I'd say make it more interactive, like we've been saying. Use more real-life stories from mums, and maybe even have other moms come in to talk about their experiences. And definitely, more opportunities for us to chat amongst ourselves and with the experts. Make it feel like a community, not just a class. That'd make a world of difference If you ask me” (MC05).*  *“Yes, I did put some questions in the chat box during the classes. But, I found that the instructors didn't always have time to address all of the questions that were submitted. There were certain topics that I felt uncomfortable discussing in a public chat especially when you don’t know people behind the cameras. You know that kind of way”? (ML01).*  *“Erm I would say the fact that we had to type in our questions into the chat box wasn't helpful to me at all, I felt a bit hesitant to ask questions at times, as it was a large group, and I didn't want to take up too much time. Plus, some questions were answered with general information, and I didn't always get the specific guidance I needed” (MG02).*  **HEALTHCARE PROFESSIONAL’S QUOTES (LACTATION CONSULTANTS /MIDWIVES).**  *“Yeah. Yeah, I'd say. In all honesty, currently, we do not have a formal feedback mechanism in place for the online classes. This is an area where we could improve” (GC01).*  *“That’s a fair point. Our current setup, mainly due to the constraints brought on by COVID-19, hasn’t allowed for as much personalized interaction as we’d like. The importance of direct support can’t be understated, especially when it comes to breastfeeding. We're aware this is an area we need to improve on, offering more intimate support structures within the limits of what’s currently safe and feasible” (GC01).*  *“We’ve tried to include real-life stories and tips, but it seems we might not have hit the mark on making those as impactful as they could be. With all the COVID adjustments, we’ve perhaps leaned too much on the clinical side of things. It’s clear we need to bring more of those personal experiences and practical advice back into the fold to make the classes as helpful and reassuring as possible” (RC01).*  *It’s a continuous process. We are constantly looking for ways and trying to enhance our online delivery methods to make the virtual experience as enriched as possible. Again…..it’s important for me to also say that there are certain aspects we cannot fully replicate in a virtual environment. For that reason, we also provide resources for additional support, like helplines and one-on-one virtual consultations, to bridge the gap as much as we can” (LC03).* |
| **Subtheme 2.1**  Incorporating Interactive Elements into Virtual Classes (e.g., Q&A sessions, feedback sessions, breakout rooms for small group discussions). | *“Well, if we could give feedback anonymously, it might encourage more of us to share honest insights about what works and what doesn't. This could really help the educators tailor the classes more closely to our actual needs and experiences. It's all about making the class as helpful and supportive as possible for everyone involved” (MC02).*  *“Yeah, yes, I would definitely be open to considering a feedback system. It would be helpful to know if there are any areas where I could improve the classes and if there are any topics that the mothers would like to see covered in more depth” (LC03).*  *“I understand that with a large class, it can be challenging to provide individual attention to every mother. However, they could offer one-on-one sessions or smaller group discussions alongside the main class. This way, mothers who have specific questions or concerns could get more personalized guidance” (MG05).*  *“Oh, there's nothing like hearing it straight from someone who's been through it all before. We've tried to sprinkle a bit of that into our programme, but maybe we've been a bit too shy about it. With all the focus on getting the official guidance right, especially with COVID, we might've lost a bit of that personal touch. It's something we're keen to bulk up on, bringing more of those real, lived experiences into the light” (LC03).*  *“There were a few chances to ask questions through a chat function, but it wasn't the same as having a real conversation. I think creating smaller groups for discussions or having sessions where we could share our experiences and fears would have made a big difference. It's important to feel like you're not going through this journey alone” (MR02).*  *“Oh, for sure. I think having some question-and-answer sessions would've made a huge difference. And maybe some breakout rooms where we could discuss with other mums in smaller groups. It would’ve felt more personal and engaging that way. Just something to make it feel like we were actually part of the class, not just onlookers” (MG02*  *"Well, it would be great if they can throw it open for mothers to participate, like asking mothers who are willing to share their thoughts or experiences to do so, I personally believe some mothers in the class would have loved to, especially those ones who it's not their first rodeo. We learn as much from each other's experiences as we do from the experts, I would say"(ML02).*  *“I didn’t like that I couldn’t ask questions directly. I found the class a bit boring in the sense that the lactation consultants were the only ones talking and mostly repeating themselves, sometimes I felt they weren’t working together because most times, one of them still repeats what the other had already taught, so ……and we only had to type in or questions in the chat box” (MG02).*  *“It's a good system, but it's not perfect, ye know? Some mums are grand with typing out their questions in the chat box, and they get immediate responses. But others might not be as comfortable with this, especially if they’re not tech-savvy. Plus, sometimes the chat can get a bit busy, and we might miss a question here and there” (GC03).* |
| **Subtheme 2.2**  Adapting Virtual Prenatal Breastfeeding Education to Reflect Individual Preferences Through Technology. | *“It might be interesting to introduce an app or a digital platform specifically designed for the class, where you can track your learning progress, set reminders for practicing techniques, or even log your own breastfeeding journey once the baby is here. Also, virtual reality scenarios that simulate real-life breastfeeding challenges could offer hands-on experience in a very innovative way” (MC01).*  *“We've a variety of resources. Traditionally, we relied heavily on face-to-face demonstrations using models, handouts, and hands-on assistance. However, since transitioning online due to Covid, we’ve started using videos, online tutorials, and interactive webinars. We also share informational articles and direct them to trusted websites that promote breastfeeding, like the HSE website” (RC01).* |
| **Theme Three**  Quality of Prenatal Breastfeeding Education. | **POSTNATAL MOTHER’S QUOTES**  *“One day, someone would say one thing about how often to feed, and then the next, someone else would say something a bit different. It was a bit confusing, like. It made me feel a bit unsure about the whole thing. I started to wonder if it was going to be more complicated than it was worth” (MR02).*  **HEALTHCARE PROFESSIONAL’S QUOTES (LACTATION CONSULTANTS /MIDWIVES).**  *- “When all staff are on the same page, mothers receive uniform advice regardless of who they speak to. This consistency helps reduce confusion and builds trust in the support system we provide. It's crucial for mothers to feel that everyone involved in their care is working together to support their breastfeeding journey” (CC02).* |
| **Subtheme 3.1**  Consistency and quality of information received. | *“Inconsistencies can arise due to varying personal experiences and training backgrounds among professionals. When mothers receive conflicting advice, it can lead to confusion and uncertainty, which might make them hesitant about breastfeeding. It's crucial that we acknowledge these concerns and strive for clearer, more unified guidance” (MLC02).*  *"I wasn’t sure of a lot of things. But then, I had a bit of a chat with some of the other mothers from the class, and realizing we were all feeling the same way was a bit of a relief. I decided to stick with my gut in the end, but it was a close call. It's a bit of a mess when you're fighting through the fog” (MG02).*  *“It's about standardizing our training. Ensuring that all healthcare professionals involved in prenatal care, not just lactation consultants but also midwives and doctors, receive the same level of training on breastfeeding guidance. This includes up-to-date research and practices that emphasize empathy and support for the mother's choice and situation” (RC03).* |
| **Theme Four**  Integrating Socio-Cultural Insights and Partner Involvement in Breastfeeding Education | **POSTNATAL MOTHER’S QUOTES**  *“It was mentioned in the online classes that partners were welcome to attend with the mothers, but it was not emphasized? It was more of a suggestion, and the instructor mentioned that it could be helpful for partners to learn about breastfeeding too. I personally invited my husband to join me, but I can see how some partners might not be interested or able to join due to scheduling conflicts” (MR03).*  *“Well, I think it would have been helpful, yeah. It's one thing for me to relay the information to my husband, but it's another for him to hear it directly from the experts, you know? Also, he might have had questions that I wouldn't be able to answer” (MG04).*  *“Well, truth be told, it might've been nice if they'd pushed a bit more for the partner to be involved. I think having him there would've given him a better understanding of what I'll be going through, so he could support me better” (MG01).*  *“You see, breastfeeding isn't a solitary task, it’s a family affair, it impacts everyone in the house. The support from the partner, and the family at large, can be critical, especially in the early days” (MC05).*  **HEALTHCARE PROFESSIONAL’S QUOTES (LACTATION CONSULTANTS /MIDWIVES).**  *“We're hoping' that by getting' partners involved early on, it'll build a stronger support network for the mammy at home. This should help mammies feel more confident in their decision to start and continue breastfeeding. Knowing they've got the full support of their partner can really take down some of the barriers to breastfeeding” (GC02).* |
| **Subtheme 4.1**  Socio-Cultural and Partner Dynamics in Breastfeeding Decisions. | *“Oh, for sure. I think if partners were made more aware of their role, it might even get them more involved in the process from the get-go. Plus, for mums, knowing that the class promotes that shared responsibility, it could be a real draw” (MR01).*  *“Well, I think he'd have a better understanding of how important breastfeeding is, not just for the baby, but for me as well. And maybe he'd be more aware of the support I'd need from him during the early stages of breastfeeding” (ML02).*  *“I would just like to emphasize that breastfeeding is a team effort, and it's important to involve partners and other family members in the process as much as possible, as they can provide invaluable support and encouragement to the mother” (RC02).*  *“Absolutely, I do believe it's important to include partners in the classes. Having partners attend the classes can have a significant positive effect on a mother's decision to breastfeed. It not only helps the partner understand the benefits of breastfeeding but also fosters a sense of teamwork and shared responsibility in the breastfeeding journey” (RM05).* |
| **Subtheme 4.2**  Breastfeeding Confidence vs. Societal Norms. | *“Oh, this is a big one for me. I believe women should feel completely comfortable breastfeeding in public. It’s a natural part of life, but in Ireland, the attitude towards it can be so unhelpful. The classes touched on this topic, but I think they could have given more practical advice on handling public breastfeeding. There's a real lack of support and understanding. People can be quite judgmental, and that needs to change.” (MG02).*  *“Absolutely, without a doubt. I mean, it’s one thing to know how to get a good latch, but it’s another to feel confident and supported to do it in public. We need to have open discussions about the cultural attitudes and how to handle negative reactions. New mothers need to know they’re not alone in this and that they have every right to feed their child wherever they need to” (ML03).*  *“Education is key, for sure. Not just for mothers but for everyone. We need to normalize breastfeedin' and make it clear that it's a natural and important part of parentin’. Campaigns, public awareness, and support from healthcare professionals can all help. And, like I said, includin' this topic more in the classes would be a great start. It’s about creatin’ a culture where mothers feel comfortable and supported, not judged” (MC01).*  *“If we're serious about promotin' breastfeedin', we have to address all aspects of it, includin' public perception. Mothers need that confidence and support. It shouldn't be somethin' they have to figure out on their own, especially not with the societal attitudes we have here” (GC01).*  *“I reckon it's partly 'cause of the stigma itself. It's like a vicious cycle. We don’t talk about it 'cause it’s uncomfortable, an' it remains uncomfortable 'cause we don’t talk about it. Plus, there's a bit of a fear of offendin' people or causin' controversy. But if we don’t tackle it head-on, nothin' will change” (LC02).*  *“Well, we need to start by normalizin' the conversation. Bring it into the classes as a regular topic. Share stories and experiences, offer practical advice on how to manage public breastfeedin', and build a community of support among mothers. And importantly, we need to educate the wider public too. If people understood more about breastfeedin' and its benefits, maybe they'd be less likely to judge” (CC02).* |
