# Supplementary material for: A qualitative study on the perspectives of prenatal breastfeeding educational classes in Ireland: Implications for maternal breastfeeding decisions
Source: PLoS One. 2024 Dec 18;19(12):e0315269. doi: 10.1371/journal.pone.0315269 (PMC11654992; doi:10.1371/journal.pone.0315269)
Supplement: Attachment — Submitted filename: Reviewers Comment and Authors Reponses.docx [file pone.0315269.s009.docx]

**Manuscript Ref. No: PONE-D-24-33587:**

**Responses to reviewers’ comments on (Revised Title) Title:** A Qualitative Study on the Perspectives of Prenatal Breastfeeding Educational Classes in Ireland: Implications for Maternal Breastfeeding Decisions.

We sincerely thank the three reviewers for their time and effort in providing detailed and helpful comments on our paper. Their suggestions have helped to sharpen our thinking and, we hope, led to a substantially improved submission.

| **Reviewer #1** **Comments** | **How and where it was addressed** |
| --- | --- |
| Authors need to explain why they did not try to comply with saturation of data? Is there any comment? | Thank you for the constructive feedback regarding data saturation in this study. I acknowledge that the initial manuscript did not articulate how data saturation was achieved, and we have incorporated a thorough explanation in the revised version to enhance clarity and methodological transparency.  **(See page 12 in the data analysis section)** |
| The exclusion criteria were not the antonym of the inclusion criteria. | Thank you for highlighting this point regarding the inclusion and exclusion criteria. As you suggested, I have revised the criteria to ensure each exclusion criterion directly opposes its corresponding inclusion criterion.  (**See page 9 Table 1: Eligibility Criteria)** |
| The length of the manuscript should be considered. | Thank you for your feedback regarding the length of the manuscript. As PLOS ONE does not impose a word count limit, the manuscript is designed to provide a comprehensive and detailed account of the study’s methodology, findings, and implications to ensure clarity and rigor. I believe the current length is necessary to efficiently convey the depth and nuances of the research. |
| **Reviewer #2** | **How and where it was addressed** |
| Well done. Although this could benefit from a substantial cut to reduce word count, as it is thorough, the qualitative methods and analysis employed were rigorous and adequately described. I have suggested a few minor revisions. | Thank you for the positive feedback on the rigor and thoroughness of the qualitative methods and analysis. I appreciate your suggestion regarding the word count and have carefully reviewed your minor revision recommendations. Given the need to provide a detailed account of the study’s methodology and findings, I believe the current length best serves the clarity and comprehensiveness of the research. I am confident that this level of detail contributes to a robust understanding of the study’s contributions. |
| **Reviewer #3** | **How and where it was addressed** |
| **Title:**  The title needs modification.  I suggest removing the phrase "and their influence on the mother's decision to breastfeed".  This statement is quantitative in nature and is not suitable for the type of study, and on the other hand, according to the nature of the findings, it is not possible to achieve this goal. | Thank you for your helpful feedback regarding the title. I understand your concern that the original title's phrase, "and their influence on the mother's decision to breastfeed," may imply a quantitative focus. To address this, I have revised the title to remove any language suggesting causation or measurable outcomes.  The revised title, **"****A Qualitative Study on the Perspectives of Prenatal Breastfeeding Educational Classes in Ireland: Implications for Maternal Breastfeeding Decisions"** reflects a qualitative exploration of the implications of prenatal breastfeeding classes on maternal breastfeeding decisions without implying a direct influence or measurable effect. This change ensures that the title aligns fully with the study's qualitative nature and aims.  **(See study title in page 1)** |
| **Abstract:**  Findings: In addition to the findings, conclusions from the findings are also presented in this section. This writing style has created ambiguity. I suggest that it be modified in terms of writing | Thank you for your feedback on the Findings section of the abstract. I understand that including interpretations and conclusions within the findings may create ambiguity. To address this, I have revised the **Findings** section of the abstract to focus solely on the observed data. Additionally, interpretations have been moved to the **Conclusion** section. This adjustment ensures a clear separation between findings and conclusions, enhancing clarity and alignment with standard abstract structure.  **Explanation of Changes:**   - Findings in Abstract: Now focus solely on observations, without suggestions or interpretations. - Conclusion in Abstract and Main Body: Both now contain the study’s interpretations, recommendations, and implications for practice.   **(See pages 2, 3 & 57)** |
| **Discussion:**  The discussion in the abstract does not discuss this study's findings. It is suggested that the discussion be modified based on the results. | Thank you for your valuable comment regarding the discussion in the abstract. I want to clarify that the abstract does not include a separate "Discussion" section. The abstract is structured with sections for Background, Methods, Results, and Conclusion to summarize the study. |
| **Introduction:**  The phrase "This phrase should be removed from the target" should be removed from the target | Thank you for your feedback regarding the **Introduction**. I appreciate your suggestion to remove a particular phrase; however, there may have been an inadvertent typo or unclear reference in the comment, as it is difficult to determine the exact phrase. |
| **Method:**  The exclusion criteria needs to be modified. Note that the withdrawal criterion includes cases where the participants are excluded from the study after entering the study. Please revise. | Thank you for your valuable comment regarding the exclusion criteria. I would like to clarify that the study did not include withdrawal criteria because no participants were withdrawn after enrolment. The exclusion criteria were designed to determine eligibility before participants entered the study. |
| **Results:**  Data analysis should be modified. Illustrative codes are not properly compared and merged, or I cannot understand their semantic relationship with sub-themes and themes. | Thank you for your feedback regarding the data analysis and illustrative codes. We have carefully reviewed and refined the themes, sub-themes, and illustrative codes to address your concerns about semantic relationships and label clarity. Specifically, we clarified the alignment between illustrative codes and sub-themes to ensure a clear and logical structure.  **(See table 4, page 21)** |
| Please use clearer labels. Or review the categories. For example, what are the illustrative codes for the sub-theme "Breastfeeding Confidence vs. Societal Norms"? | Thank you for your valuable comment. We have restructured the sub-theme "Breastfeeding Confidence vs. Societal Norms" to **"Breastfeeding Confidence in Relation to Societal Norms"** to better reflect the qualitative focus. Additionally, we refined illustrative codes to accurately represent participant perspectives and enhance their alignment with their respective sub-themes.  **(See page 46 under Theme 4)**  In addition, we have reframed the subtheme titles:   - “Impact of Unmet Educational Expectations on Breastfeeding Decision and Self-Efficacy” to “**Experiences of Unmet Educational Expectations in Shaping Breastfeeding Decisions and Self-Efficacy.”** - “The Psychological Impact of Encountering Unexpected Challenges After an Idealized Portrayal of Breastfeeding” to **“Psychological Responses to Unexpected Challenges Following an Idealized Portrayal of Breastfeeding”**   **(See pages 23 & 25, under theme one)** |
| How could you extract the theme "Quality of Prenatal Breastfeeding Education" with one sub-them "Consistency and quality of information received". | Thank you for your feedback regarding the theme "Quality of Prenatal Breastfeeding Education" and the sub-theme "Consistency and Quality of the Information Received." To enhance semantic clarity, I understand the importance of ensuring clear distinctions between theme and sub-theme titles. To address your concern, I have refined the main title of theme three to **"Consistency and Quality of Information in Prenatal Breastfeeding Education"** and revised the sub-theme title **to "Standardization and Coordination of Guidance for Enhanced Clarity."** These adjustments ensure that the main theme (theme three) accurately reflects the focus on consistent, high-quality information. At the same time, the sub-theme title highlights the specific aspects of standardization and coordination among healthcare providers.  **(See pages 39 & 40, under theme three)** |
| **Data Analysis:**  -In many of the explanations you have given for themes and sub-themes with quotations, you have used a quantitative approach that firmly describes the relationships between cause and effect. This is a major problem and needs modification.  -For example, in the phrase "postnatal mothers highlighted a gap between the educational content and the "holistic" preparation needed for a successful breastfeeding experience. Some of the postnatal mothers felt that "inadequate knowledge" about common breastfeeding challenges contributed to "unmet expectations", negatively impacted their breastfeeding self-efficacy and subsequently affected their decision to continue breastfeeding".  -Is it possible to explain this causal relationship decisively based on the experience of one or two participants? Please --Pay attention to the explanations provided for themes and sub-themes and modify similar items. | Thank you for your valuable comment regarding the manuscript's qualitative tone. You addressed the tendency for certain explanations of themes and sub-themes to suggest a quantitative cause-and-effect relationship. In response, we have carefully revised the language throughout the manuscript, particularly in sections where themes and sub-themes are explained with participant quotations **(See pages 22- 50).** For instance, in the cited example, we previously stated:  "Postnatal mothers highlighted a gap between the educational content and the 'holistic' preparation needed for a successful breastfeeding experience. Some of the postnatal mothers felt that 'inadequate knowledge' about common breastfeeding challenges contributed to 'unmet expectations,' negatively impacted their breastfeeding self-efficacy, and subsequently affected their decision to continue breastfeeding". We have reframed this to address your concern about focusing on participants' perceptions and subjective experiences without implying measurable causation. The revised passage now reads:  **"Some postnatal mothers shared that while the educational content was informative, they felt it didn't encompass the 'holistic' preparation they anticipated for a supportive breastfeeding experience. They described how 'inadequate knowledge' about common breastfeeding challenges left them with 'unmet expectations,' which they associated with a reduced sense of confidence in their ability to breastfeed and feelings of uncertainty about continuing."**  **(See page 24).** |
| **Discussion:**  -limitations  Weakness in generalizability is part of the nature of qualitative studies. For the limitations of your study, I suggest that you mention the limitations you had in conducting the study, including methodology and data analysis. Including the method of data collection and so on. | Thank you for your constructive feedback regarding the limitations section. We have revised this section to address your suggestions comprehensively, incorporating details about the limitations related to the methodology, data collection, and data analysis.  **(See pages 56 &57)** |
| **GENERAL COMMENTS IN THE MANUSCRIPT** | **RESPONSES TO COMMENTS** |
| **Table 3: Participant demographic information.**  Is black considered an ethnicity in Ireland, the same as white? | Thank you for your valuable comment. According to the Central Statistics Office (CSO) of Ireland, the categories used in my study, including "Black," "Black Irish," and "White," are indeed acknowledged as valid ethnic classifications. The CSO's guidance, which I adhered to in my research, allows for identifying various ethnic groups, reflecting Ireland's growing diversity. The 2022 Census confirmed explicitly the inclusion of individuals identifying as "Black" or of African descent, thereby legitimizing their classification as an ethnicity similar to "White." |
| The phrase “and how they influence mothers' breastfeeding decisions” should be removed. | Thank you for your attention to the language in our manuscript. Following your feedback, we have revised phrasing to avoid cause-and-effect language and ensure it aligns with a qualitative approach. For example, the phrase "how they influence mothers' breastfeeding decisions" has been rephrased to **“how these classes are perceived to inform or support mothers' breastfeeding decisions."**  **(See pages 7 & 8)** |
| **Study Setting and selection of participants**  According to the header in this section of the method, I expected to get information about where the study was conducted, but I encountered the data collection method. | Thank you for your valuable comment. The manuscript indicated, "Four tertiary hospitals in the Republic of Ireland were selected for this study." However, we did not include the specific names of the hospitals. We have now included the names of the hospitals to provide a clearer context regarding the study's setting. |
